# Developing a Hypothetical Implementation Framework of Expectations for Monitoring Early Signs of Psychosis Relapse Using a Mobile App: Qualitative Study

**DOI:** 10.2196/14366

**Published:** 2019-10-24

**Authors:** Stephanie Allan, Simon Bradstreet, Hamish Mcleod, John Farhall, Maria Lambrou, John Gleeson, Andrea Clark, EMPOWER Group, Andrew Gumley

**Affiliations:** 1 Mental Health and Wellbeing Institute of Health & Wellbeing University of Glasgow Glasgow United Kingdom; 2 Department of Psychology and Counselling La Trobe University Melbourne Australia; 3 School of Behavioural and Health Sciences Australian Catholic University Melbourne Australia

**Keywords:** psychosis, self-management, implementation science

## Abstract

**Background:**

Relapse is a common experience for people diagnosed with psychosis, which is associated with increased service costs and profound personal and familial distress. EMPOWER (Early signs Monitoring to Prevent relapse in psychosis and prOmote Well-being, Engagement, and Recovery) is a peer worker–supported digital intervention that aims to enable service users to self-monitor their mental health with the aim of encouraging self-management and the shared use of personal data to promote relapse prevention. Digital interventions have not been widely used in relapse prevention and, therefore, little is currently known about their likely implementation—both within trials and beyond.

**Objective:**

Seeking the perspectives of all relevant stakeholder groups is recommended in developing theories about implementation because this can reveal important group differences in understandings and assumptions about whether and for whom the intervention is expected to work. However, the majority of intervention implementation research has been retrospective. This study aimed to discover and theoretically frame implementation expectations in advance of testing and synthesize these data into a framework.

**Methods:**

To develop a hypothetical implementation framework, 149 mental health professionals, carers, and people diagnosed with psychosis participated in 25 focus groups in both Australia and the United Kingdom. An interview schedule informed by the normalization process theory was used to explore stakeholders’ expectations about the implementation of the EMPOWER intervention. Data were analyzed using thematic analysis and then theoretically framed using the Medical Research Council guidelines for understanding the implementation of complex interventions.

**Results:**

All groups expected that EMPOWER could be successfully implemented if the intervention generated data that were meaningful to mental health staff, carers, and service users within their unique roles. However, there were key differences between staff, carers, and service users about what facilitators and barriers that stakeholders believe exist for intervention implementation in both the cluster randomized controlled trial stage and beyond. For example, service user expectations mostly clustered around subjective user experiences, whereas staff and carers spoke more about the impact upon staff interactions with service users.

**Conclusions:**

A hypothetical implementation framework synthesized from stakeholder implementation expectations provides an opportunity to compare actual implementation data gathered during an ongoing clinical trial, giving valuable insights into the accuracy of these stakeholders’ previous expectations. This is among the first studies to assess and record implementation expectations for a newly developed digital intervention for psychosis in advance of testing in a clinical trial.

**Trial Registration:**

ISRCTN Registry ISRCTN99559262; http://www.isrctn.com/ISRCTN99559262

## Introduction

### Background

Relapse is common for many people diagnosed with schizophrenia [1]. Relapses are linked to increased disability from loss of important relationships and reduced education and employment opportunities [2]. One estimate suggests that psychotic relapse costs £10,950 (at 6 months) compared with £2532 for no relapse, with 75% of the difference in these costs coming from inpatient treatment [3]. In the United States, excess costs from relapse range from US $6033-$32,753 [4]. Commonly, relapses are preceded by so-called early warning signs (EWS) that reflect a combination of symptoms such as anxiety, depression, suspiciousness, and uniquely personal experiences. EWS-based prevention strategies assume that identifying relapse early enough enables preventative action and averts full relapse [5]. Guidelines for psychosis in both Scotland, the United Kingdom, [2] and Australia [6] recommend early signs-based strategies as crucial for relapse prevention in routine psychosis care.

Research into reliable and valid signs of relapse is essential for early intervention aimed at minimizing the harms associated with relapse [7]. A review [8] to determine the validity of early signs as predictors of relapse in people with nonaffective psychosis found that the sensitivity (correct relapse prediction by staff) ranged from 10% to 80% (median 61%), and specificity (nonrelapses correctly identified) ranged from 38% to 100% (median 81%). Therefore, existing systems used to identify EWS have an uncertain prognostic utility and may result in an unnecessary intervention that engenders fear of relapse in service users and carers [9]. Delayed help-seeking narrows the window for timely intervention [10] and can result in the use of coercive treatment measures that confirm negative expectations [11] and make disclosure of EWS more threatening for service users. Therefore, new interventions that address problems associated with help-seeking and disclosing EWS appear warranted [12].

### Early Signs Monitoring to Prevent Relapse in Psychosis and Promote Well-Being, Engagement, and Recovery Description

One emerging application of technology in mental health care is remote self-monitoring [13]. Remote self-monitoring may improve upon traditional face-to-face monitoring by allowing more regular sampling of symptoms and, potentially, earlier detection of relapse signs. EMPOWER (Early signs Monitoring to Prevent relapse in psychosis and prOmote Well-being, Engagement, and Recovery; ISRCTN99559262) aims to develop and evaluate a mobile app for use with adults who experience psychosis. The app enables routine self-monitoring for a variety of different experiences, including psychosis (eg, hearing voices and suspicious thoughts), anxiety, mood, self-esteem, and interpersonal support. Furthermore, each time people complete an app questionnaire, they receive an *EMPOWER message*, which (depending on user input) provides links to further relevant information, practical advice, or helpful quotes. The EMPOWER algorithm aims to tailor these messages to individual changes in user well-being to promote a greater sense of control over mental health and to support self-management. EMPOWER participants will use the app for an initial 28-day baseline period to identify their typical variation in personal well-being. Significant changes from baseline will be then be triaged by a clinician. Peer support workers will be involved in setting up and personalizing the daily questionnaire, alongside regular fortnightly follow-up meetings where they will support service users in using the app.

### Implementation of Digital Interventions

Digital interventions can help address clinical priorities in psychosis, such as increasing access to psychological interventions for symptoms such as paranoia [14]. However, many effective digital interventions have failed to generalize from clinical trials into clinical practice [15,16]. Owing to concerns about generalization beyond trial contexts, the UK Department of Health [17] encourages systematic implementation research to increase an understanding of how interventions are adopted or rejected. The effectiveness of interventions (including their success in reaching the target population) can be influenced by how an intervention interacts with the context in which it was implemented [18,19]. When appraising the results of a clinical trial, it can be challenging to know whether the intervention will generalize into *real-world* contexts of clinical practice. Process evaluations assess the implementation of interventions and help predict generalizability in different contexts. The Medical Research Council (MRC) framework for process evaluation [18] recommends clear descriptions of assumptions about how the intervention is expected to be implemented within a specific context. In addition, consulting multiple stakeholder groups is recommended because this can reveal across-group variance in understandings of what the intervention is and differences in assumptions about why and for whom the intervention is expected to work. Collecting data at different time points is also recommended to characterize changes in implementation factors such as participants’ attitudes toward an intervention.

Typically, the majority of implementation research on engagement with interventions has been retrospective [20]. The MRC framework for process evaluations recommends that implementation research should proactively include key stakeholders because those expected to engage with an intervention are likely to have relevant experiential knowledge, which is useful in understanding the implementation process during a trial [18]. Qualitative research carried out *during* a trial (eg, asking service users about their experiences) can aid in understanding why an intervention might work and how context affects implementation [21]. However, *befor* e interacting with an intervention, stakeholders may have pre-existing expectations regarding implementation that will shape how they interact with a planned intervention (hypothetical acceptability). Hypothetical acceptability is measured by key stakeholders’ willingness to engage with a proposed intervention and in previous trials of digital interventions for severe mental health problems actual acceptability (assessed postintervention) is typically higher than hypothetical acceptability [22].

Theory in implementation science implies some predictive capacity [23]. Typically, implementation theory aims to create conceptual tools that enable researchers to describe, identify, and explain crucial elements of the implementation process and its outcomes [24]. Developing implementation theories in advance of empirical testing provides a framework for developing predictions about how interventions will interact with the context in which they are tested. Furthermore, completing this work allows researchers to make informed predictions about what implementation barriers that might be reasonably expected [25]. One such implementation theory, normalization process theory (NPT) [25] focuses on the work that groups and individuals do when interacting with an intervention and how they make sense of it, many intervention studies have successfully utilized NPT as a framework to guide research to more fully understand the implementation process [26]. Despite the recommended involvement of patients and members of the public within implementation research [27] and widespread assumptions that consultation work can help researchers anticipate stakeholders’ needs, capacities, and priorities [28], the MRC guidelines on process evaluation [18] report substantial empirical uncertainty regarding the value of Patient and Public Involvement (PPI) work. However, stakeholders are likely to offer insights beyond the acceptability of digital interventions (eg, predicting intervention implementation barriers during testing) and arguably have a right to be involved in research, which impacts them. Adding the insight of carers, service users, and mental health staff should lead to a clearer understanding of barriers and facilitators to implementation.

To the best of our knowledge, only one other study has [29] explored staff, carers, and service users’ perspectives of acceptability and implementation of a digital intervention for psychosis before engagement. Inclusion of these stakeholders enabled potentially diverse perspectives to be integrated into system design requirements for a mobile intervention for people who were considered to have treatment-resistant schizophrenia. Although this study is in a different population, the inclusion of multiple perspectives is a strength that could be applied to the prospective investigation of stakeholder engagement with digital interventions. In addition, there is little longitudinal research comparing stakeholder predictions pre-intervention with what happens when people interact with a digital intervention. Developing implementation theories for the EMPOWER intervention based on the expectations of staff, service users, and carers within a longitudinal process evaluation will allow for the assessment of the accuracy and the changing nature of these predictions over time, potentially highlighting the value of contextual knowledge that comes from consulting with stakeholders. We anticipate that developing an a priori implementation theory derived from stakeholder consultation will enhance implementation of the intervention in the context of a clinical trial and provide meaningful data to enable later generalization into clinical practice, a clear priority for services [15,17].

This study aimed to summarize the implementation expectations expressed within focus groups by mental health staff, carers, and service users in consultation work before a clinical trial to be able to compare these with the actual experiences of implementation observed within a feasibility study.

## Methods

### Design

This study forms part of the qualitative phase conducted before a cluster randomized controlled trial for the EMPOWER intervention (ISRCTN: 99559262). The methods are reported in line with the consolidated criteria for reporting qualitative research reporting recommendations for qualitative work [30]; a full checklist can be seen in Multimedia Appendix 1. Before the start of the study, ethical approvals were provided by West of Scotland REC (16/WS/0042) and Melbourne Health (REC/15/MH/344). Managerial approval was given by National Health Service Greater Glasgow and Clyde (NHSGG&C; GN14CP229) and North Western Mental Health Services (Project Number: 2015.286). The protocol is available in the National Institute of Health Research website [31]

### Eligibility and Recruitment

All participants came from 1 health board area in the United Kingdom and 1 in Australia, where the intervention will be tested in a multisite clinical trial. Staff who support people with psychosis within Community Mental Health Services (CMHS) were invited to take part through initial researcher contact with clinical team leaders. Service user participants were invited to take part in focus groups through mental health staff and organizations providing support or representation to people with mental health difficulties. Service user participants were eligible if they were in contact with CMHS, had experienced a relapse within the previous 2 years, had received a diagnosis of Diagnostic and Statistical Manual of Mental Disorders-5 psychosis-related condition, and were able to provide informed consent. People who identified as carers for someone with psychosis were recruited from both mental health services and support organizations.

### Focus Groups

Using focus groups rather than individual interviews enabled respondents to interact with and respond to the ideas and comments of other participants with whom they shared a role [32]. Focus groups were held in private rooms (of either CMHS or support organizations) and conducted by members of the research team using a topic guide. We did not collect demographic data beyond whether the participant was a carer, service user, or mental health staff. Following best practice guidelines [18], we used an explicit theoretical framework to guide our focus group schedule. An interview schedule informed by NPT [33] was developed to explore stakeholders’ expectations. A copy of the topic guide for each of the stakeholder focus groups is provided in Multimedia Appendices 2-4.

A total of 25 focus groups were held across Melbourne and Glasgow from July 20, 2016, to September 9, 2017. Participants were 88 mental health staff, either working in the NHS in the United Kingdom (n=54, 9 focus groups) or NorthWestern Mental Health (public run) services in Australia (n=34, 4 focus groups). Focus group length ranged from 57 min to 2 hours and 9 min. A total of 21 service users were recruited from the United Kingdom (n=5, 3 focus groups) and Australia (n=16, 4 focus groups) and 40 carers from the United Kingdom (n=20, 2 focus groups) and Australia (n=20, 3 focus groups). Carers and service users received UK £20 or Aus $40 for participation. Staff received no cash reimbursement and participated during their usual working day. All participants gave written consent before taking part. All focus group facilitators (AG, SB, AC, ML, JG, JH, JF, and SA—a mix of genders) identified themselves as researchers to conduct the research and were transparent if they also held a clinical role. All participants received a presentation about the EMPOWER intervention. The focus groups were audio recorded and then transcribed verbatim. NVivo software (QSR International) was utilized to perform analysis.

### Reflexivity

SA is a Doctor of Philosophy student investigating the implementation of digital interventions for psychosis. Facilitating focus groups was a task shared by all coauthors. Data analysis was primarily completed by SA, who has previously utilized qualitative methods. Supervision and code checking for all analysis (including discussions about saturation) were provided by AG and HM, both of whom are clinical psychologists. AG is chief investigator for the EMPOWER study and was responsible for the overall design and conduct of the research.

### Data Analysis

The analysis comprised 2 stages. Thematic analysis is a qualitative method used to construct, analyze, and report on patterns within text data [34]. This is commonly utilized within qualitative aspects of process evaluations to identify key barriers and facilitators for implementation of a diverse range of digital interventions [35-37]. In stage 1, we performed an inductive thematic analysis [34] for each unique stakeholder group in turn. This was justified because in a pilot clinical trials such as EMPOWER, study evaluators are encouraged to use exploratory research to identify facilitators and barriers to interventions so that strategies can be put in place in time for an evaluation of effectiveness [18].

For stage 2, the MRC process evaluation framework [18] was identified as a suitable deductive coding framework [38] for placing the themes in an implementation theory context more relevant to the needs for a feasibility study where it may be too early to decide if normalization should be the goal. This was the rationale for moving away from our original plan (EMPOWER ISRCTN: 99559262) to use the NPT [39] framework for qualitative work. The MRC framework goes beyond barriers and facilitators to implementation and provides a taxonomy of implementation constructs. Expected barriers and facilitators (on their own) can be seen as singular aspects of a predicted overall process. However, during the analysis of focus group conversations, it was clear that barriers and facilitators were expected to *interact* together into an overall expected implementation process for EMPOWER. Therefore, we selected implementation constructs from the MRC process evaluation to structure our barriers and facilitators findings in a theoretically driven hypothetical implementation theory (presented as a deductive framework) for the EMPOWER trial:

- Reach (whether service users are expected to consent to take part)
- Fidelity (whether the intervention is expected to be used as described)
- Context (contextual factors expected to affect, or be affected by, the implementation process)
- Implementation (what successful implementation would look like in practice, beyond a trial)

**Figure 1:**
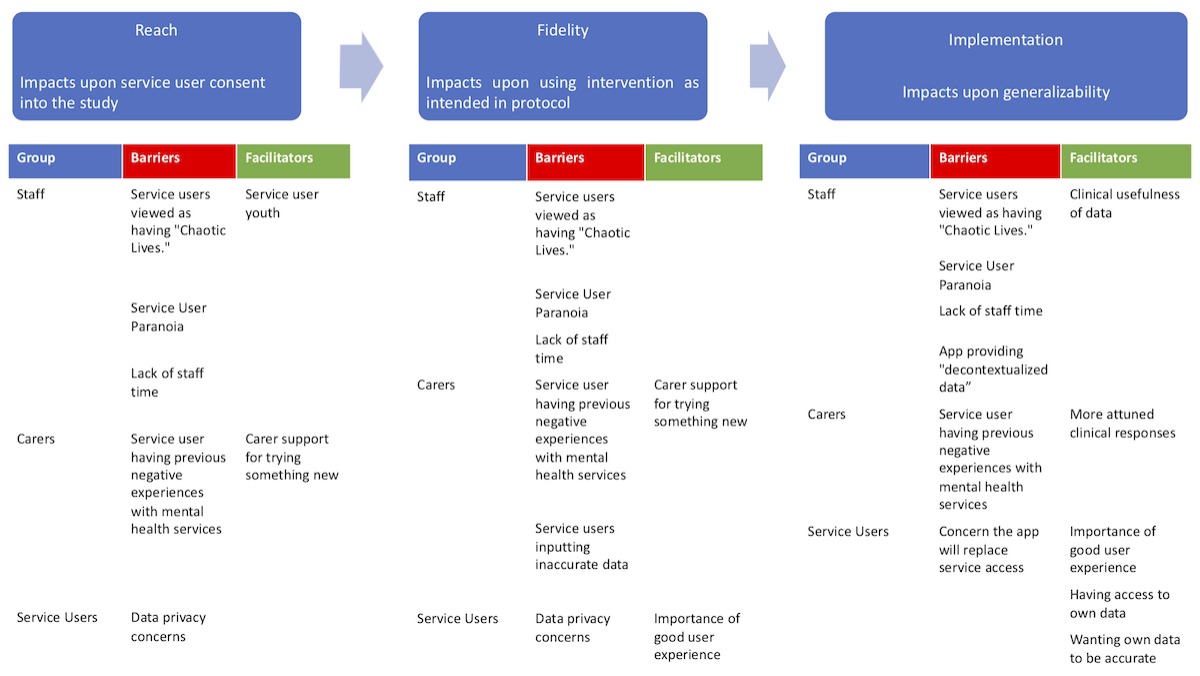
A diagram of the hypothetical implementation framework.

Coding and analyzing the data within this framework resulted in the implementation issues highlighted during inductive analysis being more meaningfully constructed as implementation barriers and facilitators. Through our initial thematic analysis, we developed 16 themes (Table 1). The implementation diagram (Figure 1) represents implementation expectations for the EMPOWER intervention across staff, service users, and carers with facilitators (green) and barriers (red) within the implementation framework. The framework analysis was completed across all stakeholder groups simultaneously.

Both stages of qualitative analysis were completed by SA and triangulated through discussion with AG and HM. Resource limitations meant that strategies such as member checking (where participants check over themes proposed by the researcher as an interpretation validity check [40]) were not utilized. However, it has been highlighted that employing this technique may increase the validity of findings in qualitative research exploring user views of digital interventions in psychosis [41] and better ensure participant views have not been misrepresented.

**Table 1:** Themes from stage 1 analysis.

Stakeholder group	Expected implementation barriers	Expected implementation facilitators
Staff	Service users viewed as having *chaotic lives*	Service user youth
	Service user paranoia	Clinical usefulness of data
	Uncertainty about whether early warning signs data are useful in early intervention services	—^a^
	App providing *decontextualized data*	—
	Lack of staff time	—
Carers	Service user having previous negative experiences with mental health services	More attuned clinical responses
	Service users inputting inaccurate data	Carer support for trying something new
Service users	Data privacy concerns	Having access to own data
	Concern the app will replace service access	Wanting own data to be accurate
	—	Importance of good user experience

^a^Some cells are empty as there were fewer themes constructed.

## Results

The first part of the Results section introduces the inductive thematic analysis (as shown in Table 1) and offers example quotes as an attempt to illustrate our analysis transparently.

### Inductive Results

#### Mental Health Staff Implementation Expectations

##### Implementation Facilitators

###### Youth

Many staff predicted that young people (eg, those accessing early intervention services for psychosis) were more natural consumers of digital interventions. Staff perceived young service users as being both familiar with and highly able to use digital technology. Staff also expected that older service users would find the intervention harder to use and to be too burdensome for this reason. These assumptions appeared commonplace throughout discussions in both the United Kingdom and Australia:

I do think it’s going to be a good thing in the long term, but there’s going to be clients that don’t fit into it now as well as. Because I think the next generation of people coming through are going to have been grown up with technology and are going to be okay with using it...

Participant 8, Staff group 11, Australia

###### Clinical Usefulness

Most staff appeared cautiously optimistic about the value of the data from the EMPOWER app and believed that it could be useful for their clinical practice by enabling staff to tune themselves into the changes in early signs and the broader context for these changes. In this particular illustrative quote, staff members highlighted how they expected EMPOWER data could draw their attention to patterns and links between stress and psychotic symptoms in the life of a service user:

You see where the stressors are, what times, what the patterns are, the patterns would be so clear.

Participant 1, Staff group 2, United Kingdom

##### Implementation Barriers

###### Service Users Viewed as Having Chaotic Lives

Staff reported that service users with a *chaotic* life would struggle to use the intervention. Staff viewed those individuals with chaotic lives as being the most vulnerable to relapse. *Chaotic lives* was a complex term referring to multiple factors including service users having difficulties with reflecting on their own experiences, having lack of insight, poor social or cognitive functioning, avoidance of services, or an inability to retain a mobile phone. These factors were considered in the context of the influence of a broader context of social deprivation or financial problems leading to users selling a provided mobile phone for cash:

It sounds like there’d be quite a specific group of patients that would benefit from this in terms of the people who are able to kind of reflect, who are you know, their lives aren’t so chaotic that they can’t keep hold of a mobile phone, you know, it doesn’t end up somewhere else or in someone else’s hands or whatever, and it’s—I think it will be really useful for people who are functioning at that level and are able to reflect on things like that, but I guess it’s—I suppose I’m just thinking it’s a shame because it’s often the people I suppose who I wonder might be at more risk of more kind of relapsing or being lost in the system somehow and becoming very unwell, are maybe already a bit too chaotic or functioning at too poor a level supposed to be able to make use of something as helpful potentially as this.

Participant 1 Staff group 7, United Kingdom

###### Service User Paranoia

Although the EMPOWER intervention was commonly described by staff to be an acceptable tool for managing relapse in at least some service users, they also perceived the intervention would not be acceptable to others. One common implementation barrier expected by staff was that service users with paranoid and/or delusional beliefs about technology would not engage with the intervention. This implementation expectation appeared grounded in expectations about how changing levels of paranoia will vary with technology affinity and competence. Conversations about service users who have technology-focused beliefs were frequent throughout staff focus groups and can be exemplified in the quote below where a staff member wonders aloud if EMPOWER would work for someone who already has such concerns about digital technology. Furthermore, this staff member highlighted that these beliefs could become more pronounced in the context of relapse:

I’m thinking about one of my service users in particular who, when he becomes unwell, his phone is actually part of his delusional belief system, and he becomes obsessive about certain part of it; so I’m wondering how that would work for him?

Participant 4, Staff group 6, United Kingdom

###### Uncertainty About Whether Early Warning Signs Data Are Useful in Early Intervention Services

Despite the optimistic expectation staff held about younger service users engaging with the intervention, staff from early intervention services discussed some different implementation barriers not present in other focus groups. For instance, the early stages of psychosis can be an uncertain time for clinicians because EWS of relapse might not be established yet. As illustrated below, a staff member from an early intervention service within the United Kingdom highlighted that the EMPOWER intervention might face a different implementation barrier because the data gathered via the app might have limited utility for staff in predicting relapse:

It’s a trial but it is quite on the edge of relapse, which is risky. With our patient group, relapse signature is not that familiar because of early on. So, you’ve not got that history to learn from.

Participant 2, Staff focus group 3, United Kingdom

###### App Providing Decontextualized Data

Many staff expressed the concern that the quantitative self-reported data gathered from service users through their usage of the app lacked the context that comes from typical interactions staff have with service users. Overall, data alone were understood as being potentially unhelpful without the clinical experience of staff members to interpret these data. Staff valued their knowledge and relationship-based experiences of service users as a basis for making decisions concerning the risk of relapse. There was an additional concern that the quantity of data could also potentially block effective decision making. An example of this can be seen below where a staff member highlighted that if information from the EMPOWER app implies that a service user is relapsing, they would not feel comfortable acting on this information alone:

a bit of an overload of information perhaps if we’re getting like you know three or whatever plus messages from the app a day and we'd need to do a management plan around...at presentation and a big limitation in that sort of context is that you don’t...it’s difficult to get a feel from the person about what is happening for the person...

Participant 3

missing out on the interpersonal context

Participant 4, Staff group 12, Australia

###### Lack of Staff Time

Staff were concerned that using the intervention in practice might be difficult. Working with people with a diagnosis of psychosis was described as a time-intensive part of their role. Staff reported having many other competing demands on their time and limited resources to do their jobs. Staff frequently referred to a lack of capacity in the system and resource constraints. Several mental health staff even reported the lack of available resources within the mental health system and were concerned that digital technologies might one day replace their jobs. In the example below, the other participants in the focus group agree with participant 1, expressing concern about the potential lack of staff capacity for the implementation of EMPOWER:

It definitely makes sense, in that my only worry about it is that thinking about my caseload at the moment and I just don’t know where we’d have the capacity to be working with it. [Sounds of Agreement from Other Participants]Particularly because it’s psychosis and schizophrenia illness and how disabling that is...erm, to people.

Participant 1, Staff group 2, United Kingdom

#### Carers’ Implementation Expectations

##### Implementation Facilitators

###### More Attuned Clinical Responses

Many carers expressed the view that routine monitoring and access to chart data could result in more attuned responses from mental health services because the data would indicate when support was needed. They believed that this would result in their loved one engaging with services when necessary, and services having a response that was experienced by their loved ones to be more relevant, timely, and acceptable. As demonstrated in the example below, carers state that they expect themselves to have a role in starting the help-seeking process:

if the chart was, you notice yourself it’s is negative, they are definitely going down the tube, you will encourage them, if they don’t see their doctor on a regular basis, that we should go and visit a doctor.

Participant 2, Carer group 3, Australia

###### Carer Support for Trying Something New

Aside from reporting implementation concerns for EMPOWER, carers also said it was essential to try out new interventions aimed at improving the lives of people with psychosis. Throughout all focus groups, it seemed clear that carers valued that clinical researchers were attempting to introduce innovation and were supportive of the role of research. Although carers were cautious about how successful their encouragement may be, they appeared keen to encourage ongoing usage of self-monitoring interventions by people who they support:

if we [as carers] had a good working understanding of it [EMPOWER] I’d find it easier to say to her “oh how are you getting on with the app?” and just encouraging her with it if she was happy to be encouraged, yeah. So, I think that’d be really good.

Participant 5, Carer group 1, United Kingdom

##### Implementation Barriers

###### Service User Having Previous Negative Experiences With Mental Health Services

However, similar to staff, carers frequently expressed that they expected the intervention to face multiple implementation barriers. Carers were nearly unanimous that the previous experiences of people with psychosis accessing services are likely to shape the reach of the intervention. This can be seen in the example below, where a carer predicts that her son is unlikely to use the EMPOWER intervention because of his previous autocratic experience dealing with mental health services. However, she remains cautiously optimistic about the implementation potential for the intervention of service users with different experiences:

I just...in my son's case, he wouldn’t use it. He just wouldn’t use it. And that’s down to the experiences he’s had with what he says is the mental health authorities. He’s really...but for people who are open to it, it would be terrific. [murmur of agreement from other participants

Participant 7, Carer group 5, Australia

###### Service Users Inputting Inaccurate Data

Carers reported widespread concern that their loved ones may inaccurately input data. Throughout focus groups, this was understood as a function of concerns that their loved ones would downplay or minimize their experiences to avoid unwanted responses and interventions from services that they believe could result from accurate data input:

I suppose in some people if they are trying to be over positive and not give the truth.

Participant 3, Carer group 1, United Kingdom

#### Service Users’ Implementation Expectations

##### Implementation Facilitators

###### Having Access to Own Data

Service users expected that having access to their data could be a useful source of learning about and becoming attuned to their well-being. Focus group discussions highlighted that psychotic experiences and general well-being are very changeable for service users. Data access appeared understood as a potential way to explore and learn about possible patterns, which might exist in these same well-being changes. In this particular example, a service user remarks that having data might encourage them to use the app because they feel that they are not currently aware of how their well-being fluctuates:

I would use them to see what’s making me happy, what’s doing my head in. How is my sleep schedule, am I getting ill. It’s just understanding your own mind better than when you’re doing it yourself. Because you’re not really aware of all these things. You forget what you done yesterday.

Participant 1, Service user group 1, United Kingdom

###### Wanting Own Data to Be Accurate

Service users reported valuing having their data and expressed an awareness that for EMPOWER to work optimally, data entry will need to be accurate. In recognition of this, service user participants reflected on the importance of responding to the survey to the best of their ability. In the example below, a participant describes this being an implementation facilitator because inaccurate data would make the app data meaningless and would not confer any benefit:

don’t lie to yourself because if you lying to the app the you are lying to yourself and basically you are not doing anyone any favours.

Participant 2, Service user group 5, Australia

###### Importance of App Providing a Good User Experience

Service users highlighted the importance of the app being appealing to use and the proposed message content being relevant and nonpatronizing. In the example below, a service user highlighted how they would feel infuriated if they were made to feel patronized. However, they stated that if they had control over what content they had to read, this would improve acceptability. Discussions such as this were commonplace and suggested that service users’ perceptions of intervention content were a vital implementation expectation:

Participant 1: Yeah. There’s a risk that it might be a wee bit patronising. Just a risk, I don’t know. I know that me personally if I was feeling down in the dumps and I got a message saying “go for a walk”...

laughs

Researcher 1: “Pull your socks up.”

Participant 1: Yeah. It may infuriate me. But maybe if I had the option to read the message, I was choosing to read the message, it wouldn’t be so annoying.

Service user group 1, United Kingdom

However, user experience conversations were not limited to intervention content. Discussions about the importance of how the app looks were common throughout focus groups. In the example below, a participant highlighted the importance of the intervention providing good experience through aesthetics. Therefore, the importance of user experience seems to envelop both intervention content as well as the package in which the intervention is delivered:

if it looks decent, if it doesn’t look like a ten-year-old made it. Yeah. It has to be engaging and it look visually... that’s pretty important to me. Not what I stand for, a ten-year-old

Participant 3, Service user group 7, Australia

##### Implementation Barriers

###### Data Privacy Concerns

Some service users stated that EMPOWER might be unacceptable to them because of expected paranoia. However, more common concerns were expressed regarding the privacy of data inputted into the app. The example below suggests that the service user is already concerned about threats to their privacy/autonomy and highlights that they are wary because their information will be sent to the treating team. Although this specific example highlights concern about information going to mental health staff, the focus group conversations also revealed concerns about other people, such as government employees or hackers, getting access to personal data. Therefore, this theme may reflect existing privacy concerns in the lives of service users. Although service users were generally accepting of the intervention regarding its role in supporting self-monitoring, they were cautious and guarded about being monitored by others, particularly mental health services:

Participant 3: We know that nothing is essentially private, well I happen to know that nothing that you tell any counsellor or social worker, nurse, therapist, anything, everything you tell them can be transferred even if it’s just in the lounge in the kitchen during lunchtime “oh blah de blah de blah.” We know they share information about us. We know they... um there is no privacy. Well I know it.

Participant 1: Uh what was the question again?

Researcher 1: It’s really about the security arrangements and confidentiality with app as we have explained it, if there is any concerns or comments about that?

Participant 3: Totally, it’s going to be sending information to the treating team

Service user group 7, Australia

###### Concern App Will Replace Service Access

Service users throughout focus groups described accessing mental health services as a source of support in managing their well-being. The EMPOWER intervention was described as likely to encounter implementation barriers if the technical side of the intervention was perceived to be replacing *high-touch* human connection. In the example below, a service user participant highlighted that the digital intervention on its own would be a poor substitute for dealing with a person who knows them:

seems a poor substitute for seeing a person that knows you

Participant 2, Service user group 1, United Kingdom

### Deductive Results

Barriers impacting upon reach (who consents to participate in the trial) are expected early in the implementation process. For example, carers expect that service users with previous negative experiences such as coercive treatment will be less likely to consent to the study (a reach barrier). Mental health staff expected that service users who have low general levels of functioning and/or high levels of paranoia would not consent or struggle to use the app if they do. However, mental health staff expected that younger service users would be more likely to be willing to participate in a digital intervention study because their generation are *digital natives*. Implementation issues that impact upon fidelity (such as service users inputting inaccurate data) are expected slightly later in the implementation process. However, even if the implementation is successful (with service users completing daily self-monitoring) and the data are perceived to be an accurate reflection of their mental state—problems in using EMPOWER data for relapse prevention are still expected. For example, staff predicted that EMPOWER data will not be applicable within the context of early intervention services because EWS of relapse will still be unclear for people experiencing first episode psychosis (an implementation barrier). Barriers such as a lack of staff time were constructed as a predicted barrier across all levels (ie, expected to impact upon everything from service user consents into a feasibility study all the way up to generalizing into clinical practice if clinical outcomes in a definitive randomized controlled trial were favorable). The results of this deductive analysis can be seen in Figure 1.

Table 1 presents the themes as barriers and facilitators constructed during the inductive analysis.

Figure 1 presents the hypothetical implementation framework that scaffolds both barriers and facilitators themes that came up during focus group discussions. The diagram shows that throughout all stages, barriers and facilitators reach, fidelity, and implementation were constructed as coming from context.

## Discussion

### Principal Findings

This study is among the first to assess and record implementation expectations across mental health staff, carers, and service users for a newly developed digital intervention for psychosis in advance of testing in a clinical trial, building on previous multistakeholder work [29]. We have identified and theoretically framed the most common implementation expectations expressed by mental health staff, service users, and carers in advance of the EMPOWER clinical trial. Understanding the context behind empirical outcomes from novel digital mental health interventions is key in deciding if an intervention can be easily implemented within current practice [16] or will require significant resources and effort to do so [42]. Within a standard implementation science approach, context is defined as a shared environment, which can provide either barriers or facilitators for implementation [18]. However, within a complexity science–informed understanding, context is defined by an intervention interacting with multiple enacted environments of different social actors [43]. Although the MRC process evaluation framework provides a theoretical framework, creating the framework shown in Figure 1 means that it is more tailored to the clinical context of relapse management as reported by carers, mental health staff, and service users. Our findings provide a complexity science–informed account of how different stakeholders expect EMPOWER to interact within the multistakeholder actions that already occur during routine relapse prevention.

Key to the proposed framework (Figure 1) is a similarity between groups regarding expectations of what would constitute successful implementation. For successful implementation, it was agreed that EMPOWER must enable service user participants to self-monitor to a level of granularity that results in data allowing for visualization of potential personal indicators of relapse while also giving a comprehensive insight into overall service user mental health. Despite this implementation expectation appearing similar across groups, there were some role differences between staff, service users, and carers. The context of health care settings is constructed as being institutionalized [44] because behaviors by social actors are described in terms of the roles people are expected to act out. Our findings suggest that implementing the use of EMPOWER data in relapse prevention is only expected to be successful if the data are symbolically meaningful [15] to each stakeholder’s role. For example, in the case of staff, this means having data that enables them to understand better how a participant feels and can help them differentiate EWS of relapse from a false alarm. For carers, useful data were constructed as staff becoming more attuned and being able to differentiate relapse signals from false alarms. Although both staff and carers emphasized data access as being an implementation facilitator that could improve service responses, service users were more curious about the impact of having access to a record of their self-reported day-to-day well-being. Previous qualitative research conducted with service users exploring potential [29,41,45] and actual [46] acceptability of digital self-management interventions for psychosis has reported that having access to personal data may have positive impacts such as enhancing self-management. However, this previous study also highlights more negative impacts reported by service users such as creating concerns about data privacy [41] and paranoia [46] and that using digital interventions may eventually lead to a reduction in mental health services [41]. Therefore, the mixed findings from our study appear mainly in line with previous research.

Similar to previous work exploring hypothetical implementation expectations held by staff, service users, and carers for a digital intervention for an online portal for schizophrenia [29], we found key differences in implementation expectations across staff, service users, and carers. Service user implementation expectations for both barriers and facilitators most frequently focused on individual experience. For example, the importance of EMPOWER providing a good user experience was highlighted as a key implementation facilitator throughout all stages of the implementation process and will be very important for sustained intervention use. User experience has been described as a neglected area within digital intervention research [47] and psychosis more specifically [48]. A recent study examining a mobile health platform for clinical monitoring in psychosis indicates that implementation was low because of the app frequently crashing [49], perhaps highlighting the importance of exploring user experience in implementation research. Carers (similar to findings from previous qualitative work [50]) and staff generally reflected how they foresee EMPOWER influencing service user interactions with staff. Furthermore, staff foreseeing digital interventions having an impact on staff roles and responsibilities is similar to previous qualitative research work conducted with mental health staff [29,51]. Carers expected that previous negative experiences of mental health care could act as a barrier toward initial engagement with the app. For carers, this expectation appeared to be related to a fear that EMPOWER would come to emulate existing dynamics within relapse prevention that can block timely communication of EWS. These findings are in line with previous research demonstrating that different stakeholders can hold different perspectives on digital mental health interventions [29,52,53] and suggest value in seeking out all relevant stakeholder perspectives.

This consultation work was helpful to the EMPOWER study because it highlighted key concerns of key stakeholders. For example, staff reporting a concern that app-generated data would be decontextualized data that may not be useful for clinical decision making. Going forward into the feasibility study, the role of a clinician in triaging data from the intervention to place app data within a meaningful context was emphasized to staff during recruitment.

### Limitations

This study has several limitations. First, focus groups may result in some participants feeling reluctant to share their views fully. Second, the implementation barriers and facilitators highlighted in this paper were those that were most commonly discussed throughout the focus groups. However, the quantity of discussion of barriers and facilitators may not equal their importance or relevance [54]. Third, participants were given a *presentation* that covered the EMPOWER rationale and how the intervention works. Participants might have formed different expectations if they were presented with an *actual* prototype. A recent recommendation for undertaking complexity science–informed implementation research within health care services is to abandon attempts to simplify implementation research but rather explore implementation more inductively from multiple perspectives [55]. Therefore, there is a concern that adopting existing implementation taxonomy from the MRC process evaluation framework [18] within our analytic approach may have overly simplified construction of the hypothetical implementation framework. Moreover, following the NPT framework in designing research questions may have minimized the range of potential responses from participants. Finally, PPI can range from consultation to stakeholders having decision making over the aims and conduct of a study [56]. Therefore, these findings should be considered in light of them coming from consultation and not direct stakeholder involvement.

### Conclusions

The field of digital self-monitoring interventions in psychosis is rapidly expanding [46,48], and there is a need to optimize interventions for implementation. One critical implementation-focused strategy is intervention co-design with stakeholders to develop digital psychosis interventions more suitable to the needs of end users [57,58]. After completion of the EMPOWER feasibility trial, we will utilize observations amassed during the trial to base comments on how stakeholder expectations identified from this analysis compare with actual trial implementation. This qualitative work done in advance of the EMPOWER trial provides insight into very early implementation expectations that form when people are first told about a digital intervention. These implementation expectations seem associated with the role that a person plays in managing a health problem (such as being a patient or a carer) as well as their previous experiences. Furthermore, these expectations extend across different levels of implementation [59], from early engagement to posttrial implementation—indicating that expectations are complex and wide ranging. Our results suggest that potential participants may quickly form implementation-related expectations about interventions and make predictions about how they (and others) will interact with the intervention. These findings indicate that potential participants do not arrive at interventions in a naïve state and may develop expectations and assumptions about new technology before they even use it for themselves.

## Appendix

Multimedia Appendix 1 Completed consolidated criteria for reporting qualitative research form.

Multimedia Appendix 2 Staff focus group schedule.

Multimedia Appendix 3 Service user focus group schedule.

Multimedia Appendix 4 Carer focus group schedule.

## Abbreviations

CMHS: Community Mental Health Services
EMPOWER: Early signs Monitoring to Prevent relapse in psychosis and prOmote Well-being, Engagement, and Recovery
EWS: early warning signs
MRC: Medical Research Council
NHS: National Health Service
NHSGG&C: National Health Service Greater Glasgow and Clyde
NPT: normalization process theory
PPI: patient and public involvement
