# Supplementary material for: Developing a Hypothetical Implementation Framework of Expectations for Monitoring Early Signs of Psychosis Relapse Using a Mobile App: Qualitative Study
Source: J Med Internet Res. 2019 Oct 24;21(10):e14366. doi: 10.2196/14366 (PMC6838692; doi:10.2196/14366)
Supplement: Multimedia Appendix 2 [file jmir_v21i10e14366_app2.pdf]

Phase 1 Topic Guide – People providing services (WP2)
Version 3: 28th July 2016

Setting Up:

1. Two facilitators required – one lead and one co-facilitator
2. Ensure consent forms signed and retained
3. Labels for people to write their first names
4. Have laptop computer and projector to stimulate discussion in Topic Areas
5. Having a flipchart and pens to aid and punctuate discussion
6. Post its available for people to write up things they would rather share individually
7. Have refreshments available for participants
8. Arrange room to ensure comfort for participants – chairs in semicircle or chairs around table
9. Arrange space for participants to access in case of distress
10. Plan for break after 1-hour

Aims: (i) To evaluate the acceptability and usability of mobile symptom recording using smartphones amongst professional mental health care staff; (ii) to identify incentives and barriers to implementation by Mental Health staff; and (iii) the identification of relapse prevention pathways and whole team responses.

What	Format	Questions	Prompts	Notes
**Introductions and welcome**	Talk	NA	NA	• Welcome<br>• Who we are<br>• Why we are here<br>• Purpose of the session<br>• Expected timings<br>• Breaks<br>• Any questions?<br>• The digital recorder and its functioning<br>• Taking timeout<br>• Informed consent
	In pairs	• *How do you currently use mobile/digital in your work supporting people?*	• *Text reminders?*<br>• *Accessing information quickly?*<br>• *Planning and organizing (e.g. diary functions, map functions)?*	• Warm up exercise – will return to topic later<br>• When discussion has dried up ask people for feedback which is written up on flipchart with any themes identified and potentially expanded upon.
**Opening discussion: establishing participants own norms**	Whole group	• *What have been your experiences of helping people prevent or minimize relapse?*	• *How is it emphasized in the team?*<br>• *How is it detected?*<br>• *Whose responsibility is it to detect risk of relapse?*	Write up points on flipchart

What	Format	Questions	Prompts	Notes
		• *Is the goal to prevent relapse or minimize impact?*	• *Is this view shared across team?*<br>• *Is this shared with users and carers?*	
		• *What are strengths of current practice?*	• *How do you respond?*<br>• *How does the team respond?*<br>• *How do users and carers respond?*	Continue to write up points on flipchart
		• *How do you know when things are starting to break down for someone you support?*	• *Change in presentation, lack of contact etc*	
		• *What is the first action you would typically take if you thought someone was becoming unwell?*		
		• *Is there a standard procedure or protocol you follow when someone is becoming unwell?*	• *Team, local and national level policy*<br>• *Are these consistently applied?*	
		• *What are the limitations of existing practice?*	• *Does it need to be improved?*<br>• *What could be improved?*	NPT Collective Action
**Introducing EMPOWER**	Slide(s)	NA	NA	Slide(s) on EMPOWER and the stepped care approach.
	Whole	• *Does the EMPOWER approach*	• *Anything which is*	NPT Coherence

What	Format	Questions	Prompts	Notes
	group	*make sense?*	*unclear?*	
		• *What advantages might the use of mobile technology bring?*	• *What might block successful use?*<br>• *How acceptable do you think it might be for people?*	NPT Coherence
		• *Do you think there could be risks associated with EMPOWER and if so what?*	• *Adverse events*<br>• *Not picking up on EWS*<br>• *Over sensitivity (false positives)*	
		• *What would help the team to successfully use EMPOWER?*	• *What would keep you involved / committed*	NPT Cognitive Participation
		• *How do you feel about the triage nurses role in EMPOWER?*		
		• *How do you feel about peer workers having a role in supporting EMPOWER users?*		
**Sharing information from EMPOWER**	Whole group	• "How do you feel about the capacity to share information and charts from the App?"	• *Any concerns?*	NPT Cognitive Participation and Collective Action
**Acting on information received from EMPOWER**	Whole group	• *Imagine you were supporting someone using the App and you were contacted by the triage nurse about a concern they had about scoring:*	• *How would you respond?*<br>• *Would this be any different to what happens now?*	NPT Cognitive Participation and Collective Action

What	Format	Questions	Prompts	Notes
			• *What opportunities or challenges could alerting bring?*	
**Overall and concluding thoughts**	Whole group	• *Thinking of EMPOWER overall and looking ahead how would you know it's worth the effort?*	• *How might you lose confidence in EMPOWER?*<br>• *How would you know if this was working/not working?*	NPT Reflexive Monitoring
**Beta testing**	Whole group	• *Would anyone be willing to test the App with someone they support?*	• *Details of beta-testing requirements and dates.*	
**Thanks and close**				
