# Supplementary material for: Developing a Hypothetical Implementation Framework of Expectations for Monitoring Early Signs of Psychosis Relapse Using a Mobile App: Qualitative Study
Source: J Med Internet Res. 2019 Oct 24;21(10):e14366. doi: 10.2196/14366 (PMC6838692; doi:10.2196/14366)
Supplement: Multimedia Appendix 3 [file jmir_v21i10e14366_app3.pdf]

Early signs monitoring to prevent relapse and promote wellbeing, engagement and recovery

Phase 1 Topic Guide – People in receipt of services (WP1)
Version 8: 9th September

Setting Up:

1. Two facilitators required – one lead and one co-facilitator
2. Ensure consent forms signed and retained
3. Labels for people to write their first names
4. Have laptop computer and projector to stimulate discussion in Topic Areas
5. Digital recorder ready and checked for functioning
6. Having a flipchart and pens to aid and punctuate discussion
7. Post its available for people to write up things they would rather share individually
8. Have refreshments available for participants
9. Arrange room to ensure comfort for participants – chairs in semicircle or chairs around table
10. Arrange space for participants to access in case of distress
11. Plan for break after 1-hour
12. Have participant expenses available for end of Focus Group

What	Format	Questions	Prompts	Notes
**Introductions and welcome**	Talk	NA	NA	• Welcome<br>• Who we are<br>• Why we are here<br>• Purpose of the session<br>• Expected timings<br>• Breaks<br>• Expenses<br>• Any questions?
**Opening discussion: establishing norms**	Whole group	• *How do you currently use mobile phones?*	• *Do you have one?*<br>• *Is it a smartphone?*<br>• *Do you use Apps?*	To explore existing practices
**Opening discussion: establishing participants own norms**	Whole group or pairs	• *How do you currently look after your mental health and wellbeing?*	• *Do you have a staying well plan?*	Facilitators should decide/enquire as to whether people might prefer working as a whole group or as pairs.
		• *How do you know when things are taking a turn for the worse in your mental health?*	• *Do you notice any changes in your ability to function (e.g., connect w family/friends),*<br>• *What you think, or how you feel, etc?*	
		• *What do you do if you think you are becoming unwell?*	• *How do you feel?*<br>• *What do you think?*<br>• *What do you do?*<br>• *What actions do you take?*	
		• *How do you feel*	• *What gets in the way?*	Continue to write up points on flipchart

What	Format	Questions	Prompts	Notes
		about getting help if you think you are becoming unwell?		
		• *Have services encouraged you to have a plan for staying well?*	• *Crisis plan, advanced statement, wellness plan?*	Continue to write up points on flipchart
**Introducing EWS monitoring**	Slide(s)	NA	NA	Covering staying well, anticipating crises, monitoring thoughts and feelings, being in the driving seat
	Whole group	• *Does this make sense?*	• *Anything which is unclear?*	NPT Coherence
		• *How helpful do you think it is to monitor early warning signs?*	NA	
**Introducing EMPOWER**	Slide(s)	NA	NA	Description of EMPOWER in terms of wellbeing monitoring and messaging to enhance control and recovery. Examples of items and process and messages. Could usefully be based on a scenario.
	Whole group	• *Does that make sense to you?*	• *Anything which is unclear?*	NPT Coherence
		• *What would help you to successfully use EMPOWER?*	• *Do you think you have the skills or experience in using a mobile phone?*<br>• *What support would you like?*<br>• *What would keep you*	NPT Cognitive Participation

What	Format	Questions	Prompts	Notes
			involved / committed	
**Sharing information and other people's involvement**	Slide(s)	NA	NA	Monitoring wellbeing and sharing wellbeing with others. What happens to the information they put into EMPOWER and who has access to it.
	Whole group	• *Does what you've just heard make sense?*	• *Concerns, question?*	NPT Coherence
		• *How would you use your data?*	• *Who would you share it with?*<br>• *When would you share it?*	NPT Cognitive Participation and Collective Action
		• *How would you like others to be involved with the App?*	• *Family and friends? Would you get help from them? Show them how it was going etc?*<br>• *Mental health staff?*	
		• *How do you feel about what happens to the information you put into the App?*	• *Confidentiality*<br>• *Suspiciousness*	
**Staged EMPOWER responses**	Slide(s)	NA	NA	Wellbeing message options and types.
	Whole group	• *What would you find helpful?*	• *Which of these would be preferable?*<br>• *Quotes, websites, videos?*	There is the potential to do this is in a more participative way with e.g. voting stickers for preferences.
		• *What type of message might be most likely to encourage you to*	NA	

What	Format	Questions	Prompts	Notes
		take action?		
		• *Would you like to help with messages?*	-	Explain opportunities for potential involvement. In both sites this could be via the online survey of message suggestions. In Glasgow there may be opportunities to play a role in validating/assessing messages.
	Slide(s)	NA	NA	Stage 2: Triage and responding to changes in wellbeing.
	Whole group	• *"Does what you've just heard make sense?"*	• *Cause anxiety?*	NPT Coherence
		• *How would this be different to what happens now?*	-	NPT Coherence (differentiation)
		• *How would you like others to respond?*	• *Family and friends?*<br>• *Mental health staff?*	NPT Collective Action
**Frequency of monitoring**	Whole group	• *How would you feel about this type of daily monitoring?*	• *Is that too often?*<br>• *What would work for you?*<br>• *Would you like to be able to vary frequency?*	
**Overall and concluding thoughts**	Whole group	• *Thinking of EMPOWER overall how would you know it's worth the effort?*	• *What might make you stop using EMPOWER?*<br>• *How would you know if this was working/not working?*	NPT Reflexive Monitoring
**Thanks and close**				
