# Supplementary material for: Developing a Hypothetical Implementation Framework of Expectations for Monitoring Early Signs of Psychosis Relapse Using a Mobile App: Qualitative Study
Source: J Med Internet Res. 2019 Oct 24;21(10):e14366. doi: 10.2196/14366 (PMC6838692; doi:10.2196/14366)
Supplement: Multimedia Appendix 4 [file jmir_v21i10e14366_app4.pdf]

EMPOWER

Early signs monitoring to prevent relapse and promote wellbeing, engagement and recovery

Phase 1 Topic Guide – Carers (WP1)
Version 3: 21st September, 2016

Setting Up:

1. Two facilitators required – one lead and one co-facilitator
2. Ensure consent forms signed and retained
3. Labels for people to write their first names
4. Have laptop computer and projector to stimulate discussion in Topic Areas
5. Digital recorder ready and checked for functioning
6. Having a flipchart and pens to aid and punctuate discussion
7. Post its available for people to write up things they would rather share individually
8. Have refreshments available for participants
9. Arrange room to ensure comfort for participants – chairs in semicircle or chairs around table
10. Arrange space for participants to access in case of distress
11. Plan for break after 1-hour
12. Have participant expenses available for end of Focus Group

What	Format	Questions	Prompts	Notes
**Introductions and welcome**	Talk	NA	NA	• Welcome<br>• Who we are<br>• Why we are here<br>• Purpose of the session<br>• Expected timings<br>• Breaks<br>• Expenses<br>• Any questions?
**Opening discussion: establishing participants own norms**	Whole group or pairs	• *How do you support your family member/friend to look after their mental health and wellbeing?*	NA	• Facilitators should decide/enquire as to whether people might prefer working as a whole group or as pairs.<br>• Facilitators to write points of flip chart.
		• *How do you know when things are taking a turn for the worse?*	• *What do you notice? (E.g., changes in their ability to function, what they say and/or do, etc.)*	
		• *What do you do if you think your family*	• *What actions do you take? For example,*	

		member/friend is becoming unwell?	*contacting key clinician or crisis service, monitoring medication, talking things over, etc.*	
		• *How do you feel about seeking the support of a professional (e.g., contacting key clinician) if your family member/friend is becoming unwell?*	• *What gets in the way?*	
		• *Have services encouraged your family member/friend to have a plan for staying well?*	• *Crisis plan, advanced statement, wellness plan?*	
**Introducing EWS monitoring**	Slide(s)	NA	NA	Covering staying well, anticipating crises, monitoring thoughts and feelings, being in the driving seat
	Whole group	• *Does this make sense?*	• *Anything which is unclear?*	NPT Coherence
		• *What are your experiences of EWS monitoring?*	• *Have you been encouraged to monitor your family*	

			member/friend's EWS, or to help them monitor their EWS? If so, by whom?<br>• *What EWS do you look for?*<br>• *Advantages and disadvantages*	
		• *How helpful do you think it is to monitor EWS?*	NA	
**Introducing EMPOWER**	Slide(s)	NA	NA	Description of EMPOWER in terms of wellbeing monitoring and messaging to enhance control and recovery. Examples of items and process and messages. Could usefully be based on a scenario.
	Whole group	• *Does that make sense to you?*	• *Anything which is unclear?*	NPT Coherence
		• *What role would you like to play in introducing your family*	• *Informing identification of idiosyncratic EWS and or coping*	NPT Cognitive Participation and Collective Action

		member/friend to the App?	*strategies;*<br>• *Support of baseline monitoring;*<br>• *Offering technical expertise*	
		• *If your family member/ friend was using the EMPOWER App, how would you like to be involved?*	• *Providing reminders for them to use the App;*<br>• *Helping them to use the App;*<br>• *Contributing to the information that they enter (e.g., agreeing on scores)*	NPT Cognitive Participation and Collective Action
		• *What are the benefits to active carer involvement with the EMPOWER App?*	•	NPT Cognitive Participation and Collective Action
		• *What are the disadvantages of active carer involvement with the EMPOWER App?*	• *Confidentiality*<br>• *Suspiciousness*	NPT Cognitive Participation and Collective Action
**Sharing information**	Slide(s)	NA	NA	Monitoring wellbeing

**and other people's involvement**				and sharing wellbeing with others. What happens to the information their family member/ friend enters into EMPOWER and who has access to it.
	Whole group	• *Does what you've just heard make sense?*	• *Concerns, questions?*	NPT Coherence
		• *How would you like your family member/friend to use their data?*	• *Who (e.g., you, key clinician, treating team, etc.) would you like them to share it with?*<br>• *How often would you like to see their charts?*<br>• *How likely do you think it is that they will share data?*	NPT Cognitive Participation and Collective Action
**Staged EMPOWER responses**	Slide(s)	NA	NA	Wellbeing message options and types.
	Whole group	• *What messages would you value?*	• *Which of these would be preferable: quotes, websites, and/or videos?*	There is the potential to do this is in a more participative way with e.g. voting stickers for preferences.

		• *Would you like to help with messages?*	-	Explain opportunities for potential involvement. In both sites this could be via the online survey of message suggestions. In Glasgow there may be opportunities to play a role in validating/assessing messages.
	Slide(s)	NA	NA	Stage 2: Triage and responding to changes in wellbeing. Including an overview of the role of carers/ next of kin.
	Whole group	• *Does what you've just heard make sense?*	-	NPT Coherence
		• *How would this be different to what happens now?*	-	NPT Coherence (differentiation)
		• *What do you think about the triage nurses role/ NWMH's role in EMPOWER?*	-	NPT Collective Action

**Overall and concluding thoughts**	Whole group	• *Thinking of EMPOWER overall how would you know it's worth the effort?*	• *What might make you stop using EMPOWER?*<br>• *How would you know if this was working/not working?*	NPT Reflexive Monitoring
**Thanks and close**				
